# Blood pressure and vascular determinants of glomerular filtration rate decline in diabetic kidney disease

**DOI:** 10.3389/fcvm.2023.1230227

**Published:** 2023-07-27

**Authors:** Luca Truscello, Dina Nobre, Vehashini Sabaratnam, Olivier Bonny, Grégoire Wuerzner, Michel Burnier, Fadi Fakhouri, Menno Pruijm, Anne Zanchi

**Affiliations:** ^1^ Service of Nephrology and Hypertension, Department of Medicine, Lausanne University Hospital and University of Lausanne, Lausanne, Switzerland; ^2^ Service of Endocrinology, Diabetes and Metabolism, Lausanne University Hospital and University of Lausanne, Lausanne, Switzerland

**Keywords:** diabetic kidney disease, GFR decline, blood pressure, pulse wave velocity, renal resistive index, carotid intima-media thickness

## Abstract

**Objective:**

In patients with type 2 diabetes and diabetic kidney disease (DKD), explore the relationship between estimated glomerular filtration rate decline (eGFR-d) and simultaneously assessed vascular risk markers including office, ambulatory or central blood pressure, pulse pressure, carotid-femoral pulse wave velocity (PWV), carotid intima-media thickness (IMT) and renal resistive indexes (RRI).

**Research design and methods:**

At baseline, vascular risk markers were measured in addition to the routine clinical workup. The eGFR-d was based on 2000–2019 creatinine values. Parameters were compared by eGFR-d quartiles. Regression models of eGFR-d and vascular markers were assessed.

**Results:**

In total, 135 patients were included. Mean age was 63.8 ± 10.8y, baseline eGFR 60.2 ± 26.4 ml/min/1.73 m^2^ and urine albumin-creatinine ratio (ACR) 49 ± 108 mg/mmol. Mean eGFR-d was based on 43 ± 39 creatinine values within a time span of 7.0 ± 1.9y. The average yearly eGFR decline was −1.8 ± 3.0 ml/min/1.73 m^2^ ranging from −5.8 ± 2.3 in the first quartile to +1.4 ± 1.7 in the fourth quartile. Mean 24 h systolic (SBP) and diastolic (DBP) blood pressure were 126 ± 17 and 74 ± 9 mmHg. Mean PWV was 11.8 ± 2.8 m/s, RRI 0.76 ± 0.07 and IMT 0.77 ± 0.21 mm. SBP and pulse pressure correlated with eGFR-d but not DBP. 24 h SBP stood out as a stronger predictor of eGFR-d than office or central SBP. PWV and RRI correlated with eGFR decline in univariate, but not multivariate regression models including 24 SBP and ACR.

**Conclusions:**

In this study, eGFR decline was highly variable in patients with type 2 diabetes and DKD. Twenty-four hour SBP provided an added value to the routine measurement of ACR in predicting eGFR decline, whereas PWV and RRI did not.

## Graphical Abstract

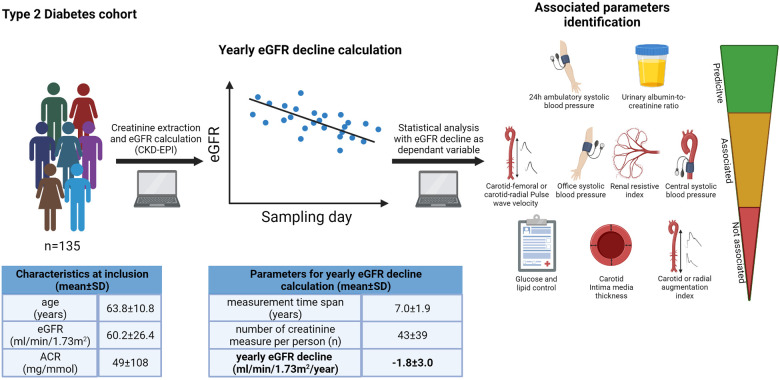

## Introduction

Diabetic kidney disease (DKD) affects 30%–40% of diabetic patients and is the main cause of end-stage kidney disease (ESKD) in developed countries (1). The rate of decline of renal function is highly heterogeneous (2). Classical biomarkers such as albuminuria are associated with adverse renal outcomes, but more than 30% of DKD patients do not have albuminuria and still progress to ESKD (3). Finding new biomarkers is crucial for the early identification of those at highest risk of accelerated renal function decline.

Better glycemic control, renin-angiotensin system blockade and recently SGLT2 inhibitors, GLP1 receptor agonists and finerenone are the standard of care for type 2 diabetic patients at high cardio-renal risk (4). Landmark randomized controlled trials (RCT) with empagliflozin (5), canagliflozin (6), dapagliflozin (7) and finerenone (8) improved cardio-renal outcomes when prescribed to patients with proteinuric DKD. All these drugs have hemodynamic effects, suggesting that vascular factors have an important role in disease progression. Furthermore, elevated blood pressure, pulse pressure, pulse wave velocity, intima media thickness and renal resistive index have all been associated with eGFR decline (eGFR-d); however, the relative contribution of each factor to eGFR-d in the same individual has not been explored in detail, due to a lack of studies that have measured these vascular markers simultaneously (9–12).

The Swiss Diabetic Nephropathy Cohort (SWIDINEP) (*N*CT03407989) is a cohort of type 2 diabetic patients with DKD stage G1-G4 followed at the Lausanne University Hospital (CHUV) in a prospective and longitudinal way with detailed vascular phenotyping at standardized time points. In this study, we explore the relationship between eGFR decline and a panel of vascular markers measured at enrollment including blood pressure and its level of control, pulse pressure, pulse wave velocity, intima-media thickness and the renal resistive index.

## Methods

Patients with type 2 diabetes (T2D) were recruited at the kidney and diabetes outpatient clinics of the Lausanne University Hospital between 2013 and 2019. Both males and females were enrolled with a diagnosis of DKD defined by at least one of the following criteria: an eGFR <60 ml/min/1.73 m^2^, albuminuria >30 mg on a 24 h urine collection or an ACR >2.5 mg/mmol for men or >3.5 mg/mmol for women measured in at least two urinary samples. Age over 18 years and the ability to read and understand the consent form were required for inclusion. All participants followed the standardized protocol of investigations in our outpatient clinic. In case there was the slightest doubt about the presence of another underlying kidney disease, they were excluded from this cohort, and referred for further nephrological workup. Patients were excluded from the study if other causes of kidney disease were present (apart from hypertensive nephropathy, focal segmental glomerulosclerosis, obesity glomerulomegaly and nephrolithiasis). All had a renal ultrasound examination. Hence, post-renal causes of kidney disease were identified thanks to renal ultrasound, as were patients with polycystic kidney disease, and these patients were not enrolled in the cohort. Eligible patients were asked to return the consent form duly signed before inclusion in the cohort.

Patients included in the cohort underwent a complete check-up onsite. Medical history, clinical and paraclinical data, sociodemographic status as well as current treatments and smoking status were recorded. A complete set of clinical data such as patient's height, weight, body mass index (BMI), seated blood pressure (BP) and heart rate were measured after 15 min rest using a brachial cuff-based oscillometric device (OMRON HEM-907). Routine serum and urine chemistries were collected including renal function, glycemic control and lipid profile. A complete vascular evaluation was proposed: (1) 24 h ambulatory BP was measured (ABPM, Diasys, Physicor, Geneva Switzerland; validated by a minimum of 20 measurements during daytime and 7 measurements during nighttime) (13). Percentage dipping was defined as the difference between daytime mean systolic BP and nighttime mean systolic BP expressed as a percentage of the day value. (2) Arterial stiffness and central BP were assessed non-invasively with the commercially available Sphygmocor device (Version 8.0, At Cor Medical, Sydney, Australia) using applanation tonometry to measure respectively carotid-femoral pulse wave velocity (PWV) and carotid and radial augmentation indexes. Patients were supine for at least 10 min before measurements. For details, we refer to a locally performed, previous study (14). (3) Carotid intima media thickness was measured with an ultrasound system [Aplio XG device (Toshiba Sytems, AG/Sa Switzerland)] in the supine position after at least 5 min rest (15). Images of the longitudinal and transverse planes of the internal carotid artery were obtained. Mean carotid artery thickness was measured in the longitudinal plane with dedicated software in an area free of plaques, 10 mm proximal of the carotid bifurcation, at the end of diastole in both the right and left common carotid artery. The bulbus was excluded from IMT measurements. (4) Renal ultrasound of both kidney was performed with the same ultrasound system and included renal dimensions and measurement of renal resistive indexes on 3 segmental arteries (superior, middle, and inferior) in each kidney. Imaging data and waveform analyses were performed by respectively the same medical doctor (M.P.) and research nurse (D.N.), both with large experience in each technique, according to previously published protocols (14).

All kidney function data were extracted on site from patient medical records. The CKD-EPI formula was used for eGFR calculation (16). For each patient, eGFR was calculated using every creatinine values available from years 2000 to 2019. From these eGFR values, linear regression was performed with the day of the sample as independent variable and the eGFR as dependent variable. The resulting line was then plotted to obtain the absolute change in kidney function in the last ten years. This method provided for each patient a single value representing the yearly eGFR decline. If a patient were to enter renal replacement therapy (RRT) during the study, the linear regression was calculated up to the date of RRT. Patients with less than four eGFR values or a time span of less than 666 days were excluded from the analysis. All linear regressions, lines and equations were performed using the commercially available software Microsoft Excel 32 bites (Microsoft Corporation, Washington) with built-in functions. Analysis of factors influencing eGFR decline slopes was based on clinical and biochemical parameters collected at baseline.

### Statistics

Unless stated otherwise, results are expressed as mean ± SD. Normality was assessed with the Shapiro-Wilk test and visually with boxplots. Outliers were defined as values higher than 2.5 times the mean value. Analyses of variance (ANOVA) was used to evaluate statistical significance for continuous variables with normal distribution; Kruskal-Wallis non-parametric test was used to evaluate statistical difference for not normally distributed variables. Post-hoc analyses were performed with Tukey's methods for ANOVA and Dunn's test using Sidak correction method for Kruskal-Wallis non-parametric test. For frequency variables, multiple chi squared tests were used to evaluate statistical differences. For correlation analysis, Pearson correlation coefficient method was used followed by linear regression analysis with the adjusted R^2^ reported in Results; adj *R*^2^ refers to the amount of variance in eGFR explained by the parameter of interest in univariate linear regression analysis. A threshold of *p* < 0.05 was set for statistical significance. The total sample size required for a correlation coefficient of 0.3 was of 68. A multivariate stepwise regression analysis was run to identify the best predictors of eGFR slopes. This model specified the significant level of 0.05 for entering in the model and 0.1 for removing it from the model. All statistical analyses were performed with the commercially available STATA 16 software (Stata college station, Texas).

### Data protection and ethics

The SWIDINEP study was submitted and approved by the Cantonal Human Being Ethics Research Committee (CER 43/12), the Centre Hospitalier Universitaire Vaudois (CHUV) and all data were treated anonymously according to the Swiss law of data protection. The study is registered at clinical trials gov (NCT03407989).

## Results

### Baseline demographic and clinical characteristics

A total of 150 patients were enrolled in the entire cohort and data of 135 patients were available for the analysis of eGFR slopes (Supplementary Flow chart). The demographic and clinical characteristics of these 135 patients are described in Tables 1, 2. Mean age was 63.8 ± 10.8 years with a predominance of Caucasians (83%) and males (76.3%). Average BMI was 31.6 ± 5.3 kg/m^2^ with respectively 7.4%, 35.6%, 35.6%, 15.6% and 5.9% with normal weight, overweight and obesity stage I, II and III. Active smoking was present in 23.7% of patients with 50.4% and 25.9% former smokers and non-smokers. Women were significantly heavier than men but smoked less (Supplementary Table S1). Mean eGFR was 60.2 ± 26.4 ml/min/1.73 m^2^ and urine ACR ratio was of 48.9 ± 108.4 mg/mmol. Half of patients were KDIGO stage A2 (ACR: 3–29 mg/mmol) and 26.8% were KDIGO stage A3 (ACR ≥ 30 mg/mmol).

**Table 1:** Clinical characteristics of patients enrolled in SWIDINEP at the baseline visit.

	%	Mean	SD
Age (y)		63.8	10.8
Sex			
Male	76.3		
Female	23.5		
Ethnicity			
Caucasian	83		
Asian	1.5		
African	8.2		
Other	7.4		
Duration of diabetes (y)		13.3	9.0
Diagnosis of hypertension	96		
Duration of hypertension (y)		12.3	20.9
BMI (kg/m^2^)		31.6	5.3
Smoking History			
Past	50.4		
Active	23.7		
Never	25.9		
Past medical history			
Coronary artery disease	35.8		
Stroke	9.0		
Peripheral artery disease	23.0		
Retinopathy	35.7		
Proliferative retinopathy with laser therapy	37.3		
Peripheral neuropathy	54.8		
Erectile dysfunction	51.0		
Lower limb amputation	4.9		
Renal function			
eGFR (CKD-EPI) ml/min/1.73 m^2^		60.2	26.4
KDIGO CKD stage			
1	17.1		
2	29.1		
3a	18.7		
3b	25.4		
4	9.7		
eGFR decline (ml/min/1.73 m^2^/y)		−1.8	3.0
Albumine/creatinine ratio (mg/mmol)		48.9	108.4
KDIGO CKD stage			
A1	22.0		
A2	51.2		
A3	26.8		

**Table 2:** eGFR decline quartiles and clinical characteristics.

	All	1st quartile	2nd quartile	3rd quartile	4th quartile	*p* value	Post hoc
*n*	135	34	37	31	33		
eGFR decline (ml/min/1.73 m^2^/year)	−1.8 ± 3.0	**−5.8 ± 2.3**	**−1.9 ± 0.5**	**−0.6 ± 0.4**	**+1.4 ± 1.7**	**0**.**0001**	^*,†,‡,§,||,#^
min		−11.1	−3.0	−1.1	+0.04		
max		−3.1	−1.2	0.0	+7.0		
Fast decliners (%) ^a^	12.6%	50%					
Average Time Range days (years)	2,545 ± 706 (7.0)	2,378 ± 681 (6.5)	2,597 ± 761 (7.1)	2,779 ± 543 (7.6)	2,437 ± 764 (6.7)	0.1	
Number of creatinine measured	42.5 ± 39.0	**60.2 ± 46.8**	**32.8 ± 27.7**	**34.9 ± 27.8**	**42.2 ± 44.8**	**0**.**006**	^*,†,‡^
eGFR at entry (ml/min/1.73 m^2^/year)	60.2 ± 26.4	**49 ± 23.7**	**61.1 ± 26.4**	**66.9 ± 28.6**	**64.7 ± 24.6**	**0**.**02**	^†,‡^
min	15	16	15	23	22		
max	129	98	112	129	108		
ACR ratio (mg/mmol)	49 ± 108	**100 ± 141**	**63 ± 139**	**16 ± 24**	**10 ± 10**	**0**.**0001**	^†,‡^
min	0	3	0	0	0		
max	694	477	694	93	45		
Age (y)	64 ± 11	66 ± 9	65 ± 12	62 ± 12	62 ± 10	0.2	
Sex (%)						0.5	
men	76	85	73	71	76		
women	24	15	27	29	24		
Weight	91.5 ± 18.8	92.0 ± 14.9	88.0 ± 17.1	92.2 ± 22.6	93.6 ± 20.7	0.8	
BMI	31.6 ± 5.3	31.4 ± 3.7	31.3 ± 4.9	32.0 ± 6.5	31.7 ± 6.2	0.9	
Smoking history (%)						0.9	
Past	50.4	47.1	54.1	51.6	48.5		
Active	23.7	20.6	24.3	22.6	27.3		
Never	25.9	32.4	21.6	25.8	24.2		

^a^ >5 ml/min/year.

^*^ Post-hoc analysis for *p*-value < 0.05 between the first and second quartile.

^†^ Post-hoc analysis for *p*-value < 0.05 between the first and third quartile.

^‡^ Post-hoc analysis for *p*-value < 0.05 between the first and fourth quartile.

^§^ Post-hoc analysis for *p*-value < 0.05 between the second and third quartile.

^||^ Post-hoc analysis for *p*-value < 0.05 between the second and fourth quartile.

^#^ Post-hoc analysis for *p*-value < 0.05 between the third and fourth quartile.

Bold significants ANOVA with *p* < 0.05.

The majority of patients were on anti-hypertensive therapy (96%); RAS blockade (87.8%) and diuretics (thiazide or loop; 66.2%) were the most prescribed antihypertensive drugs. Other therapies such as calcium channel blockers (43.2%) and beta-blockers (41.2%) were less prescribed. Mineralocorticoid-receptor antagonists (8.1%), alpha-blockers (3.4%) and peripheral vasodilators (3.4%) were rarely used.

Regarding T2D medication, the majority of patients were on metformin (56.4%) or insulin (59.4%) followed by a DPP-IV inhibitor (36.8%). Only 15.8% of patients were on a GLP1R agonist and 6.8% on an SGLT2 inhibitor at baseline.

### Renal function decline

The time span for the estimation of eGFR decline was an average of 2,545 ± 706 days (7.0 ± 1.9 years) based on an average of 43 ± 39 creatinine values per patient. Globally, the average yearly change in eGFR was −1.8 ± 3.0 ml/min/1.72 m^2^/year ranging from a decrease of −11.1 to an increase of 7 ml/min/1.73 m^2^/year. A trend to a lesser decline was observed in women compared to men (−1.25 vs. −1.9 ml/min/1.73 m^2^/year (Supplementary Table S1) but the difference was not significant. Fast eGFR decline as defined by an eGFR decrease > 5 ml/min/1.73 m^2^/year was present in 17 subjects (12.6%, 16 males, 1 female) (Table 2).

Table 2 describes the quartiles of eGFR decline and their clinical characteristics. The yearly eGFR decline ranged between −5.8 ± 2.3 ml/min/1.73 m^2^/year in the first quartile to +1.4 ± 1.7 ml/min/1.73 m^2^/year in the fourth quartile. Age, sex, weight, BMI and smoking history did not differ between quartiles. The number of creatinine measurements was higher in the first quartile and baseline eGFR at entry of the cohort was lower in the first quartile compared to the last quartile. There was a remarkable wide range of baseline eGFR in all quartiles from KDIGO stage G1 to G4. Baseline eGFR correlated only weakly with eGFR decline (adj *R*^2^ 0.05; *p* = 0.005). The ACR ratio was significantly higher for quartiles 1 in comparison to quartiles 3 and 4 and ACR correlated with eGFR decline (adj *R*^2^ 0.09; *p* = 0.0004). Age, sex, BMI and smoking history were not correlated with eGFR decline.

### Vascular and biochemical factors and eGFR decline

#### Blood pressure (treatment, office, ABPM)

The number of prescribed anti-hypertensive classes was significantly higher in the first quartile (3.5 ± 1.3), compared to the second (2.2 ± 1.2), third (2.6 ± 1.4) and fourth quartile (2.5 ± 1.4) of eGFR decline, *p* = 0.0004.

Office systolic and diastolic BP were significantly higher in the first quartiles (Table 3). Diurnal and nocturnal systolic BP were higher in the first quartile as well as pulse pressure. Office, 24 h, day and night systolic BP correlated with eGFR decline (resp. adj *R*^2^ 0.11; *p* = 0.0001; adj *R*^2^ 0.08; *p* = 0.001, adj *R*^2^ 0.07; *p* = 0.003, adj *R*^2^ 0.08; *p* = 0.001). Likewise, office, 24 h, day and night pulse pressure correlated with eGFR decline (resp. adj *R*^2^ 0.09; *p* = 0.0002; adj *R*^2^ 0.08; *p* = 0.001; adj *R*^2^ 0.09; *p* = 0.0008, adj *R*^2^ 0.08; *p* = 0.002). Diastolic BP and mean BP did not correlate with eGFR decline.

**Table 3:** eGFR decline quartiles and office, ambulatory blood pressure measurements.

	All	1st quartile	2nd quartile	3rd quartile	4th quartile	*p* value	Post hoc
Office systolic blood pressure	136 ± 20	146 ± 21	134 ± 21	131 ± 18	131 ± 13	0.02	^*,†,‡^
Office diastolic blood pressure	75 ± 10	78 ± 10	72 ± 9	75 ± 11	75 ± 10	0.05	
Office pulse pressure	61 ± 17	**68 **± **18**	**63 **± **20**	**57 **± **14**	**56 **± **14**	**0**.**03**	^†,‡^
Office pulse rate	72 ± 11	72 ± 10	74 ± 11	72 ± 12	72 ± 10	0.5	
24 h systolic blood pressure	126 ± 17	**134 **± **19**	**125 **± **17**	**121 **± **13**	**124 **± **16**	**0.04**	^†,^
24 h variation coefficient	12.7 ± 3.7	12.8 ± 4.1	12.7 ± 3.3	13.1 ± 4.3	12.5 ± 3.7	0.9	
24hTIR%	57.8 ± 31.5	50.7 ± 32.6	53.0 ± 33.4	71.5 ± 27.9	59.2 ± 29.0	0.1	
24 h diastolic blood pressure	74 ± 9	75 ± 9	72 ± 9	75 ± 10	73 ± 9	0.4	
24 h pulse pressure	53 ± 14	**58 **± **16**	**54 **± **14**	**47 **± **9**	**51 **± **14**	**0.02**	^†,^
24 h pulse rate	77 ± 11	76 ± 9	80 ± 11	75 ± 13	79 ± 10	0.4	
Dipping (%)	−9.0 ± 8.2	−7.8 ± 8.9	−7.9 ± 7.8	−10.6 ± 8.8	−10.2 ± 7.3	0.5	
Diurnal systolic blood pressure	129 ± 17	**137 **± **17**	**128 **± **18**	**124 **± **14**	**128 **± **17**	**0.03**	^†,^
Day variation coefficient	11.9 ± 4.2	11.4 ± 4.3	12.2 ± 4.3	11.9 ± 4.6	12.0 ± 3.6	0.8	
Diurnal TIR% (<135 mmHg)	60.9	51.3	57.9	72.2	62.9	0.1	
Diurnal diastolic blood pressure	76 ± 9	78 ± 7	74 ± 10	77 ± 10	77 ± 10	0.4	
Diurnal pulse pressure	53 ± 14	**59 **± **15**	**55 **± **14**	**47 **± **10**	**51 **± **15**	**0.01**	^†,^
Diurnal pulse rate	80 ± 12	78 ± 9	83 ± 13	79 ± 13	81 ± 11	0.5	
Nocturnal systolic blood pressure	119 ± 20	**128 **± **25**	**119 **± **19**	**112 **± **14**	**115 **± **17**	**0.04**	^†,^
Night variation coefficient	11.2 ± 3.9	12.1 ± 4.4	11.1 ± 3.6	11.4 ± 3.0	10.0 ± 4.2	0.3	
Nocturnal TIR% (<120mmHg)	55.3	45.1 ± 35.9	49.3 ± 34.3	70.7 ± 27.2	60.3 ± 35.4	0.06	
Nocturnal diastolic blood pressure	68 ± 12	71 ± 12	67 ± 11	69 ± 12	67 ± 9	0.3	
Nocturnal pulse pressure	51 ± 16	**57 **± **20**	**53 **± **14**	**43 **± **8**	**48 **± **15**	**0.02**	†
Nocturnal pulse rate	71 ± 11	72 ± 9	73 ± 11	69 ± 13	71 ± 9	0.6	

^*^ Post-hoc analysis for *p*-value <0.05 between the first and second quartile.

^†^ Post-hoc analysis for *p*-value <0.05 between the first and third quartile.

^‡^ Post-hoc analysis for *p*-value <0.05 between the first and fourth quartile.

Bold significants ANOVA with *p* < 0.05.

**Figure 1:**
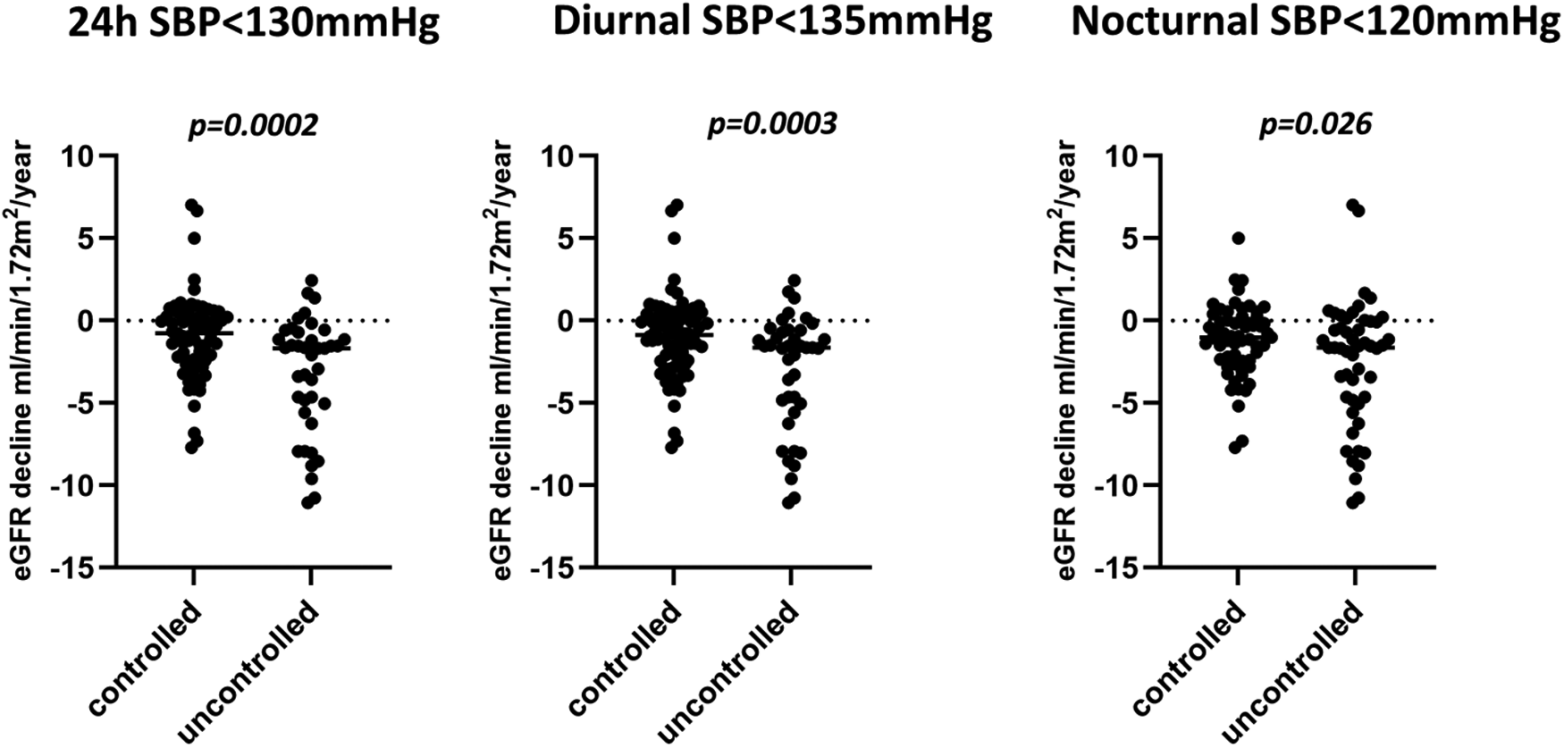
eGFR decline in patients with controlled or uncontrolled systolic blood pressure.

***Blood pressure goal attainment* (**Figure 1**):** patients with a 24 h systolic BP control <130 mmHg (63%) declined less than others (ml/min/1.73 m^2^/y: −1.0 ml ± 2.6 vs. −3.3 ± 3.5, *p* = 0.0002). Patients reaching a diurnal systolic BP control <135mmHg declined less than others (ml/min/1.73 m^2^/y: −1.0 ml ± 2.5 vs. −3.1 ± 3.5, *p* = 0.003). Patients reaching a nocturnal systolic BP control < 120 mmHg declined less than others (ml/min/1.73 m^2^/y: −1.2ml ± 2.2 vs. −2.5 ± 3.8, *p* = 0.03).

***Blood pressure variability and time in range* (**Table 3**)**: Blood pressure variability as defined by 24 h, diurnal or nocturnal variation coefficient did not differ between quartiles. Systolic diurnal and nocturnal time in range (TIR) defined as the % of time spent in range (diurnal <135 mmHg, nocturnal < 120 mmHg) did not significantly differ between quartiles although there was a trend for lower TIR for the first quartile.

***Dippers*** were defined as having at least 10% decrease in nocturnal systolic BP compared to diurnal systolic BP. Nocturnal dipping was present in 46.7% of cases with an average eGFR decline of −1.6 ml/min/1.73 m^2^/y which was non statistically lower than in non-dippers (−2.1 ml/min/1.73 m^2^/y).

#### Vascular parameters (central BP, carotid augmentation index, pulse wave velocity, carotid intima-media thickness), renal ultrasound and resistive index

Central systolic BP was significantly higher in the first quartile, as well a central pulse pressure and both correlated with eGFR decline (resp. (adj R^2^ 0.08; *p* = 0.006 and adj R^2^ 0.06; *p* = 0.02). Carotid and radial augmentation indices were similar among quartiles. Carotid-femoral pulse wave velocity was higher in the first quartile and correlated with eGFR decline (adj *R*^2^ 0.07; *p* = 0.006). Carotid-radial pulse wave velocity did not differ between groups. Left and right carotid intima-media thickness did not differ between groups. Renal volume, number of cysts measured by ultrasound were the same in all quartiles. Average left and right resistive index was significantly higher in the first quartile and correlated with eGFR decline (adj *R*^2^ 0.08; *p* = 0.001) (Table 4).

**Table 4:** eGFR decline quartiles, sphygmocor measurements, carotid intima-media thickness and renal ultrasound.

	All	1st quartile	2nd quartile	3rd quartile	4th quartile	*p* value	Post hoc
*N*							
Central blood pressure (mmHg)	*n* = 81						
Systolic	131 ± 20	**142 ** **± ** **20**	**127 **± **23**	**127 **± **21**	**126 **± **11**	**0.009**	^*,‡^
Diastolic	74 ± 13	77 ± 10	69 ± 10	75 ± 11	74 ± 13	0.09	
Pulse pressure	57 ± 18	**65 **± **16**	**57 **± **21**	**53 **± **18**	**51 **± **13**	**0.01**	^†,‡^
Augmentation index (%)							
Carotid artery	26.3 ± 11.6	27.7 ± 11.1	23.8 ± 11.2	25.4 ± 12.6	29.1 ± 11.6	0.4	
Radial artery	25.1 ± 12.4	26.6 ± 10.4	28.0 ± 11.1	23.3 ± 13.1	22.5 ± 14.2	0.2	
Pulse wave velocity (m/s)	*n* = 90						
Carotid-femoral PWV	11.8 ± 2.8	13.2 ± 2.8	12.1 ± 2.5	10.9 ± 3.0	11.4 ± 2.6	0.06	
Carotid-Radial PWV	8.2 ± 1.4	8.1 ± 1.5	8.3 ± 1.2	8.1 ± 1.5	8.1 ± 1.4	0.9	
Carotid Intima-media thickness (mm)	*n* = 121						
Left carotid (mean)	0.77 ± 0.21	0.76 ± 0.19	0.80 ± 0.22	0.75 ± 0.26	0.76 ± 0.16	0.8	
Right carotid (mean)	0.76 ± 0.21	0.80 ± 0.21	0.77 ± 0.23	0.72 ± 0.22	0.76 ± 0.18	0.5	
Renal ultrasound	*n* = 131						
Average renal volume (cm^3^)	161 ± 55	166 ± 54	166 ± 71	163 ± 42	149 ± 45	0.7	
Average resistive index	0.76 ± 0.07	**0.79 **± **0.06**	**0.78 **± **0.08**	**0.73 **± **0.06**	**0.75 **± **0.08**	**0.007**	^†,#^
Average cysts (*n*)	1.7 ± 1.6	1.4 ± 1.2	2.0 ± 2.1	2.1 ± 1.8	1.3 ± 0.9	0.7	

^*^ Post-hoc analysis for *p*-value < 0.05 between the first and second quartile.

^†^ Post-hoc analysis for *p*-value < 0.05 between the first and third quartile.

^‡^ Post-hoc analysis for *p*-value < 0.05 between the first and fourth quartile.

^#^ Post-hoc analysis for *p*-value < 0.05 between the third and fourth quartile.

Bold significants ANOVA with *p* < 0.05.

#### Biochemical values and diabetes control

Blood glucose control as defined by HbA1C was not different between quartiles. The prescription of drugs did not differ between quartiles of eGFR decline. All other biochemical variables in Supplementary Table S2 were the same in the four quartiles. As for linear regression analysis, sodium had a positive correlation with eGFR decline (adj R^2^ 0.03; *p* = 0.047). Potassium had a negative correlation with eGFR decline (adj *R*^2^ 0.04; *p* = 0.02). None of the other variables correlated with eGFR decline (Supplementary Table S3).

#### Univariate and multivariate regression analysis

Details of the univariate regression analysis for factors significantly associated with eGFR decline is presented in Supplementary Table S3. A multivariate stepwise regression analysis was run to identify the best predictors of eGFR decline from various systolic BP readings (24 h SBP, office SBP and central SBP). This model specified the significant level of 0.05 for entering in the model and 0.1 for removing it from the model. From this model, 24 h SBP stood out with the highest predictor of eGFR decline (*p* < 0.0001, R^2 ^= 0.25).

Further models explored whether other clinical parameters were additive to the 24 h systolic BP prediction of eGFR decline. A multiple stepwise regression analysis using the same criteria as above evaluated the effects of 24 h systolic BP, ACR, femoral PWV, mean renal resistive index, baseline eGFR. Two variables (24 h SBP (*p* = 0.001) and ACR (*p* = 0.01)) remained in the model as significant and independent predictors of eGFR decline. This model predicted 40% of eGFR decline variability (*p* < 0.0001).

Finally, when age, BMI and sex were included as variables in the linear regression analysis along with 24hSBP and ACR, there was no alteration in the significance or coefficients of 24hSBP and ACR. This confirms that age, BMI and sex have no impact on the relationship between eGFR decline and 24hSBP and ACR.

## Discussion

Vascular markers have been linked to eGFR decline and to morbidity but most studies focused on individual markers, without exploring whether their predictive values were equal or additive (9–12). In this cohort of 135 DT2 patients with different degrees of DKD we show that office, 24 h, and central systolic BP, pulse pressure, pulse wave velocity and renal resistive index all correlated with eGFR decline in univariate models, whereas diastolic BP, intima media thickness did not. However, the only vascular variable that remained significantly associated with eGFR decline in addition to ACR in multivariable regression models was 24 h SBP.

In this study, eGFR decline was based on the retrospective analysis of an average of 43 creatinine values over a time span of 7 years. It showed an average decline of −1.8 ml/min/1.73 m^2^/year. Fast decliners (<−5 ml/min/1.73 m2/year) represented 12.3% of cases. The rate of decline was superior to the average annual eGFR decline (−0.528 ml/min/1.73 m^2^) of 189 CKD patients of similar age of the Swiss based Colaus cohort (CKD: all causes combined, 12.7% with diabetes) (17). SWIDINEP confirms the finding of the Colaus study that diabetes is an important risk factor for a faster eGFR decline.

Office, central, diurnal and nocturnal systolic BP correlated with eGFR decline but accounted individually for only 7%–11% of its variability. Diastolic BP was not associated with eGFR decline. This relationship is in accordance with the recent KNOW-CKD study from Korea showing that office systolic BP but not diastolic BP was significantly associated with a >50% eGFR decline in CKD patients (32.4% with diabetes) followed for an average of 7.5 years (18). Another study included CKD patients (41% with diabetes) with ABPM and followed for an average of 4.7 years. This study showed that 24 h systolic BP, proteinuria and hemoglobin values were associated with renal outcomes (19). As in our study, eGFR decline was not significantly different in dippers in comparison to non dippers.

In a multivariate analysis, the 24 h systolic BP was the most strongly associated with eGFR decline in comparison to other measurements of systolic BP. In particular, measuring central systolic BP, a surrogate marker of arterial stiffness, did not add to the predictive value of 24 h systolic BP. Furthermore, the association was independent from baseline eGFR showing that it is important at all stages of DKD.

As for other surrogate markers of arterial stiffness, pulse pressure or augmentation index did not correlate with eGFR decline. Although, carotid-femoral pulse wave velocity was negatively associated with eGFR decline and with baseline eGFR in univariate analysis, it did not add to the predictive value of 24 h systolic BP and urine albumin creatinine ratio. Thus we were not able to reproduce previous findings that arterial stiffness independently predicts renal outcomes in patients with diabetes (20). This is maybe due to the fact that previous studies did not systematically take 24 h BP into account.

A recent study showed that in younger type 2 diabetic patients (<60y) with an eGFR ≥45 ml/min, aortic pulse wave velocity (PWV) predicted a decline in eGFR before the onset of advanced renal dysfunction (21). Our study was not large enough to address this issue. However, 24 h BP was not measured in the study by Fountoulakis et al. In our cohort, PWV was higher in the first and second quartiles; when stratifying patients by PWV over or under 10.3 m/s, average eGFR decline differed significantly (−0.4 ± 1.4 vs. −2.2 ± 3.1 ml/min/1.73 m^2^, *p* = 0.007). Besides, PWV exceeded on average 10.3 m/s, a threshold that was associated with a 30% increased risk of ESKD in comparison to a PWV <10.3 m/s in the CRIC cohort (22). Once more, 24hBP was not available in the CRIC cohort, hampering definite conclusion. Whether PWV measurement will improve the stratification of patients at risk beyond systolic BP and urine ACR ratio remains uncertain and needs to be explored in larger studies.

We could not show any correlation with other markers of arterial stiffness such as carotid intima-media thickness or carotid and radial augmentation indexes. The relationship between carotid-intima thickness and eGFR decline remains conflicting (23–25). A recent study in T2D shows no independent relationship between radial augmentation index and eGFR decline in T2D (26).

As for renal ultrasound, the renal resistive index alone predicted eGFR decline but accounted only for 8% of its variability. Studies are scarce on the association of renal resistive index and eGFR decline. One study examining the dynamic changes in renal resistive index with glyceryl trinitrate, showed that T2D patients with larger changes in renal resistive index has a higher risk in developing microalbuminuria after 4.1 years of follow-up (27). Another study showed a clear association between severe interstitial fibrosis, eGFR decline and renal resistive index in CKD patients due mainly to glomerulonephritis (28). Bigé et al. defined a cutoff value of ≥0.65 to identify patients with the highest risk. In our study, the vast majority (92%) had a renal resistive index ≥0.65. In another recent study, Davis et al. defined a cutoff of 0.7 but could not show an association with eGFR decline (only a minority were diabetic). When using this cutoff value in our study, 80% had a renal resistive index over 0.7 with a significantly faster eGFR decline than others (−2.2 ± 0.3 vs. −0.6 ± 0.5 ml/min/1.73 < m^2^, *p* = 0.03). Finally when using the cutoff of 0.8 as in a study of patients with T2D by Nosadini et al., 30.3% had a renal resistive index over 0.8, and these patients declined more than others (−2.8 ± 0.5 vs. −1.5 ± 0.3 ml/min/1.73 < m^2^, *p* = 0.03) (29). Our study confirms the association with eGFR decline, irrespective of baseline eGFR. However, as for PWV, RRI had no added value to predict eGFR decline on top of urine ACR and 24 h SBP. Further studies are therefore needed to identify the best cut-off value and whether this parameter adds to the stratification of patients at risk of accelerated eGFR decline and whether therapeutic interventions that decrease RRI alter the risk of eGFR decline. A pilot study showed an improvement in renal resistance index in T2D patients treated for 2 days with dapagliflozin but not with hydrochlorothiazide (30). In a recent study, we could not show any change in renal resistive index in healthy normotensive volunteers treated with empagliflozin for one month (31).

This study has several limitations. The eGFR slope was analyzed retrospectively and was based on linear regression analysis. Non-linear trajectories, episodes of acute kidney injury or changes in the prescription of drugs with renal effects were not taken into account in the analysis. GFR was estimated, thus less precise than measured GFR with iohexol or inulin clearances. However, this study examined the kinetics of eGFR with an average of 40 values of eGFR per patient. Performing a similar study with measurement GFR is not feasible. Furthermore a recent study showed that measured GFR performed only modestly better than eGFR CKD-EPI in the prediction of mortality in CKD (32). Because kidney biopsies were not performed, we cannot exclude that we included erroneously some patients with another associated kidney disease. No hard endpoints were considered in the analysis, and the slope of eGFR decline used as a hard endpoint is still a matter of debate (33). A recent study however showed that eGFR decline is associated with mortality in American Indians (34). The low number of individuals in this study decreased the power to identify weaker associations in particular in normoalbuminuric individuals. Also, patients in our cohort were in average close to blood pressure and HbA1C goals, thus these results cannot be generalized to less well controlled patients with DKD. Finally, this study did not examine the dynamic changes in vascular markers and their relationship to eGFR decline. These relationships will be examined at completion of the cohort.

The clear strength of our study is the extensive panel of vascular parameters that was performed in most participants at baseline, which allowed us to analyze their added value on top of classic risk factors, as well as their mutual correlations.

In conclusion, in this study, we demonstrate that eGFR decline is highly variable in patients with type 2 diabetes and diabetic kidney disease. Although individually associated with eGFR decline, pulse wave velocity or renal resistive index did not add to the predictive value of 24 h systolic blood pressure and urine albumin creatinine ratio. Twenty-four hour ambulatory blood pressure measurement provides an added value to the routine measurement of albumin creatinine ratio in identifying patients with type 2 diabetes at risk of accelerated eGFR decline.

## Data Availability

The raw data supporting the conclusions of this article will be made available by the authors, without undue reservation.

## References

[1] Afkarian M Sachs MC Kestenbaum B Hirsch IB Tuttle KR Himmelfarb J Kidney disease and increased mortality risk in type 2 diabetes. J Am Soc Nephrol. (2013) 24:302–8. 10.1681/ASN.2012070718 23362314 PMC3559486

[2] Shahbazian H Rezaii I. Diabetic kidney disease; review of the current knowledge. J Renal Inj Prev. (2013) 2:73–80. 10.12861/jrip.2013.24 25340133 PMC4206005

[3] Kramer HJ Nguyen QD Curhan G Hsu CY. Renal insufficiency in the absence of albuminuria and retinopathy among adults with type 2 diabetes mellitus. JAMA. (2003) 289:3273–7. 10.1001/jama.289.24.3273 12824208

[4] de Boer IH Khunti K Sadusky T Tuttle KR Neumiller JJ Rhee CM Diabetes management in chronic kidney disease: a consensus report by the American diabetes association (ADA) and kidney disease: improving global outcomes (KDIGO). Kidney Int. (2022) 102:974–89. 10.1016/j.kint.2022.08.012 36202661

[5] The EMPA-KIDNEY Collaborative Group; Herrington WG Staplin N Wanner C Green JB Hauske SJ Empagliflozin in patients with chronic kidney disease. N Engl J Med. (2022) 388(2):117–27. 10.1056/NEJMoa2204233 36331190 PMC7614055

[6] Perkovic V Jardine MJ Neal B Bompoint S Heerspink HJL Charytan DM Canagliflozin and renal outcomes in type 2 diabetes and nephropathy. N Engl J Med. (2019) 380(24):2295–306. 10.1056/NEJMoa1811744 30990260

[7] Heerspink HJL Stefansson BV Correa-Rotter R Chertow GM Greene T Hou FF Dapagliflozin in patients with chronic kidney disease. N Engl J Med. (2020) 383:1436–46. 10.1056/NEJMoa2024816 32970396

[8] Bakris GL Agarwal R Anker SD Pitt B Ruilope LM Rossing P Effect of finerenone on chronic kidney disease outcomes in type 2 diabetes. N Engl J Med. (2020) 383:2219–29. 10.1056/NEJMoa2025845 33264825

[9] Sedaghat S Mattace-Raso FU Hoorn EJ Uitterlinden AG Hofman A Ikram MA Arterial stiffness and decline in kidney function. Clin J Am Soc Nephrol. (2015) 10:2190–7. 10.2215/CJN.03000315 26563380 PMC4670762

[10] Piko N Bevc S Ekart R Petreski T Vodosek Hojs N Hojs R. Diabetic patients with chronic kidney disease: non-invasive assessment of cardiovascular risk. World J Diabetes. (2021) 12:975–96. 10.4239/wjd.v12.i7.975 34326949 PMC8311487

[11] Townsend RR. Arterial stiffness in CKD: a review. Am J Kidney Dis. (2019) 73:240–7. 10.1053/j.ajkd.2018.04.005 29908694 PMC6450550

[12] Sheen YJ Lin JL Li TC Bau CT Sheu WH. Peripheral arterial stiffness is independently associated with a rapid decline in estimated glomerular filtration rate in patients with type 2 diabetes. BioMed Res Int. (2013) 2013:309294. 10.1155/2013/309294 24471138 PMC3891544

[13] Stergiou GS Palatini P Parati G O'Brien E Januszewicz A Lurbe E 2021 European society of hypertension practice guidelines for office and out-of-office blood pressure measurement. J Hypertens. (2021) 39:1293–302. 10.1097/HJH.0000000000002843 33710173

[14] Pivin E Ponte B Pruijm M Ackermann D Guessous I Ehret G Inactive matrix gla-protein is associated with arterial stiffness in an adult population-based study. Hypertension. (2015) 66:85–92. 10.1161/HYPERTENSIONAHA.115.05177 25987667

[15] Efremov L Yang WY Jacobs L Thijs L Kuznetsova T Struijker-Boudier HAJ Post-processing reproducibility of the structural characteristics of the common carotid artery in a Flemish population. Artery Res. (2017) 19:9–17. 10.1016/j.artres.2017.04.007 28868090 PMC5567409

[16] Michels WM Grootendorst DC Verduijn M Elliott EG Dekker FW Krediet RT. Performance of the Cockcroft-Gault, MDRD, and new CKD-EPI formulas in relation to GFR, age, and body size. Clin J Am Soc Nephrol. (2010) 5:1003–9. 10.2215/CJN.06870909 20299365 PMC2879308

[17] Guessous I Ponte B Marques-Vidal P Paccaud F Gaspoz JM Burnier M Clinical and biological determinants of kidney outcomes in a population-based cohort study. Kidney Blood Press Res. (2014) 39:74–85. 10.1159/000355779 25011916

[18] Lee JY Park JT Joo YS Lee C Yun HR Yoo TH Association of blood pressure with the progression of CKD: findings from KNOW-CKD study. Am J Kidney Dis. (2021) 78:236–45. 10.1053/j.ajkd.2020.12.013 33444666

[19] Ida T Kusaba T Kado H Taniguchi T Hatta T Matoba S Ambulatory blood pressure monitoring-based analysis of long-term outcomes for kidney disease progression. Sci Rep. (2019) 9:19296. 10.1038/s41598-019-55732-4 31848394 PMC6917780

[20] Prenner SB Chirinos JA. Arterial stiffness in diabetes mellitus. Atherosclerosis. (2015) 238:370–9. 10.1016/j.atherosclerosis.2014.12.023 25558032

[21] Fountoulakis N Thakrar C Patel K Viberti G Gnudi L Karalliedde J. Increased arterial stiffness is an independent predictor of renal function decline in patients with type 2 diabetes Mellitus younger than 60 years. J Am Heart Assoc. (2017) 6. 10.1161/JAHA.116.004934 28360227 PMC5533009

[22] Townsend RR Anderson AH Chirinos JA Feldman HI Grunwald JE Nessel L Association of pulse wave velocity with chronic kidney disease progression and mortality: findings from the CRIC study (chronic renal insufficiency cohort). Hypertension. 2018;71:1101–7. 10.1161/HYPERTENSIONAHA.117.10648 29712736 PMC6342478

[23] Manabe S Kataoka H Mochizuki T Iwadoh K Ushio Y Kawachi K Maximum carotid intima-Media thickness in association with renal outcomes. J Atheroscler Thromb. (2021) 28:491–505. 10.5551/jat.57752 32759541 PMC8193787

[24] Seo DH Kim SH Song JH Hong S Suh YJ Ahn SH Presence of carotid plaque is associated with rapid renal function decline in patients with type 2 diabetes Mellitus and normal renal function. Diabetes Metab J. (2019) 43:840–53. 10.4093/dmj.2018.0186 30877715 PMC6943261

[25] Han L Bai X Lin H Sun X Chen XM. Lack of independent relationship between age-related kidney function decline and carotid intima-media thickness in a healthy Chinese population. Nephrol Dial Transplant. (2010) 25:1859–65. 10.1093/ndt/gfp718 20048122

[26] Liu JJ Liu S Lee J Gurung RL Yiamunaa M Ang K Aortic pulse wave velocity, central pulse pressure, augmentation index and chronic kidney disease progression in individuals with type 2 diabetes: a 3- year prospective study. BMC Nephrol. (2020) 21:359. 10.1186/s12882-020-02024-z 32819303 PMC7441695

[27] Bruno RM Salvati A Barzacchi M Raimo K Taddei S Ghiadoni L Predictive value of dynamic renal resistive index (DRIN) for renal outcome in type 2 diabetes and essential hypertension: a prospective study. Cardiovasc Diabetol. (2015) 14:63. 10.1186/s12933-015-0227-y 25994303 PMC4445506

[28] Bige N Levy PP Callard P Faintuch JM Chigot V Jousselin V Renal arterial resistive index is associated with severe histological changes and poor renal outcome during chronic kidney disease. BMC Nephrol. (2012) 13:139. 10.1186/1471-2369-13-139 23098365 PMC3531254

[29] Nosadini R Velussi M Brocco E Abaterusso C Carraro A Piarulli F Increased renal arterial resistance predicts the course of renal function in type 2 diabetes with microalbuminuria. Diabetes. (2006) 55:234–9. 10.2337/diabetes.55.01.06.db05-0881 16380498

[30] Solini A Giannini L Seghieri M Vitolo E Taddei S Ghiadoni L Dapagliflozin acutely improves endothelial dysfunction, reduces aortic stiffness and renal resistive index in type 2 diabetic patients: a pilot study. Cardiovasc Diabetol. (2017) 16:138. 10.1186/s12933-017-0621-8 29061124 PMC5654086

[31] Zanchi A Pruijm M Muller ME Ghajarzadeh-Wurzner A Maillard M Dufour N Twenty-Four hour blood pressure response to empagliflozin and its determinants in normotensive non-diabetic subjects. Front Cardiovasc Med. (2022) 9:854230. 10.3389/fcvm.2022.854230 35391843 PMC8981729

[32] Boele-Schutte E Gansevoort RT. Measured GFR: not a gold, but a gold-plated standard. Nephrol Dial Transplant. (2017) 32:ii180–4. 10.1093/ndt/gfw441 28158649

[33] Levey AS Inker LA Matsushita K Greene T Willis K Lewis E GFR Decline as an end point for clinical trials in CKD: a scientific workshop sponsored by the national kidney foundation and the US food and drug administration. Am J Kidney Dis. (2014) 64:821–35. 10.1053/j.ajkd.2014.07.030 25441437

[34] Suchy-Dicey AM Zhang Y McPherson S Tuttle KR Howard B Umans J Glomerular filtration function decline, mortality, and cardiovascular events: data from the strong heart study. Kidney360. (2021) 2:71–8. 10.34067/KID.0000782020 33954294 PMC8096185

