# Supplementary material for: Blood pressure and vascular determinants of glomerular filtration rate decline in diabetic kidney disease
Source: Front Cardiovasc Med. 2023 Jul 27;10:1230227. doi: 10.3389/fcvm.2023.1230227 (PMC10413385; doi:10.3389/fcvm.2023.1230227)
Supplement: Supplementary file 1 [file Presentation1.pptx]

## Slide 1
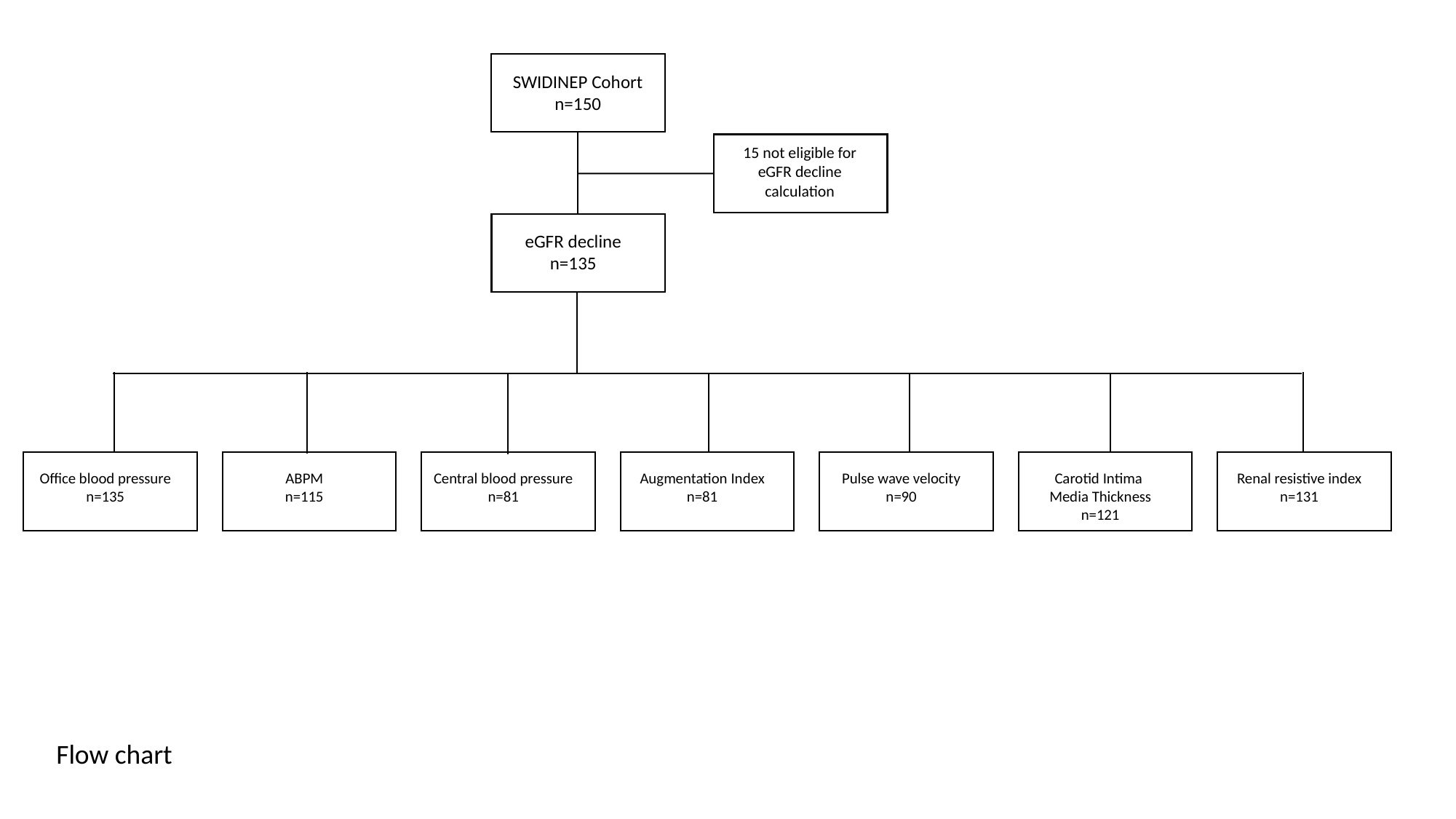

SWIDINEP Cohort
n=150
15 not eligible for eGFR decline calculation
eGFR decline
n=135
Office blood pressure
n=135
ABPM
n=115
Central blood pressure
n=81
Augmentation Index
n=81
Pulse wave velocity
n=90
Carotid Intima
Media Thickness
n=121
Renal resistive index
n=131
Flow chart
