# Supplementary material for: Blood pressure and vascular determinants of glomerular filtration rate decline in diabetic kidney disease
Source: Front Cardiovasc Med. 2023 Jul 27;10:1230227. doi: 10.3389/fcvm.2023.1230227 (PMC10413385; doi:10.3389/fcvm.2023.1230227)
Supplement: Supplementary file 2 [file Datasheet1.docx]

**Supplementary Tables**

**Supplementary Table 1. Renal function and gender differences.**

|  | Male | Female | T-test p value |
| --- | --- | --- | --- |
| n | 103 | 32 |  |
| eGFR decline | -1.9±3.3 | -1.25±1.64 | 0.2 |
| Age | 64.7±10.0 | 60.7±12.3 | 0.06 |
| BMI | 30.9±4.9 | 33.8±6.0 | 0.005 |
| TAS_24h | 127.3±18.1 | 121.3±11.6 | 0.15 |
| Duration of type 2 diabetes | 13.2±8.7 | 13.8±10.0 | 0.8 |
| Duration of hypertension | 7.6±25.1 | 19.4±9.4 | 0.1 |
| Smoke |  |  |  |
| Past | 55.3% | 34.4% |  |
| Active | 26.2% | 15.6% |  |
| Never | 18.5% | 50.0% | chi2: p=0.002 |

**Supplementary Table 2. Biochemical values by eGFR decline quartiles**

|  | All | 1st quartile | 2^nd^ quartile | 3rd quartile | 4th quartile | p value |
| --- | --- | --- | --- | --- | --- | --- |
| Ldl cholesterol (mmol/l) | 2.2±1.1 | 2.4±1.2 | 1.9±0.9 | 2.4±1.3 | 2.1±1.0 | 0.5 |
| Hdl cholesterol(mmol/l) | 1.2±0.5 | 1.1±0.4 | 1.1±0.3 | 1.3±0.7 | 1.2±0.4 | 0.9 |
| Total cholesterol (mmol/l) | 4.4±1.3 | 4.2±1.3 | 4.1±1.2 | 4.6±1.3 | 4.6±1.3 | 0.2 |
| Triglycerides (mmol/l) | 2.5±1.8 | 2.4±1.6 | 2.4±2.0 | 2.4±1.3 | 2.5±2.4 | 0.7 |
| HbA1C (%) | 7.8±1.3 | 8.0±1.4 | 7.9±1.2 | 7.8±1.3 | 7.4±1.4 | 0.4 |
| Sodium (mmol/l) | 140±3 | 139±3 | 139±4 | 141±2 | 140±4 | 0.08 |
| Potassium (mmol/l) | 4.3±0.5 | 4.4±0.5 | 4.5±0.6 | 4.3±0.5 | 4.2±0.5 | 0.05 |
| Urate (μmol/l) | 421±117 | 422±128 | 447±125 | 434±123 | 373±87 | 0.7 |
| HCO3- (mmol/l) | 22.6±3.1 | 21.4±3.0 | 23.0±3.6 | 23.3±1.6 | 23.5±3.6 | 0.2 |
| Urea (mmol/l) | 10.2±6.7 | 10.5±3.8 | 10.8±5.1 | 8.2±3.5 | 11.1±11.6 | 0.2 |
| Corrected calcium (mmol/l) | 2.3±0.1 | 2.3±0.1 | 2.3±0.1 | 2.3±0.1 | 2.3±0.1 | 0.9 |
| Phosphate (mmol/l) | 1.0±0.2 | 1.1±0.2 | 1.0±0.1 | 1.0±0.2 | 1.0±0.2 | 0.1 |
| Hemoglobin(g/l) | 136±19 | 137±18 | 132±22 | 144±15 | 134±18 | 0.08 |

Supplementary Table 3: **Pearson correlation coefficient and univariate linear regression analysis of vascular factors significantly associated with eGFR decline. Coefficients for retained parameters in the stepwise regression analysis**

|  | **Pearson correlation coefficient** | **Univariate linear regression analysis** | | | **Stepwise regression analysis** |
| --- | --- | --- | --- | --- | --- |
|  |  | Coefficient | adj R^2^ | p value | Coefficient |
| **Systolic blood pressure** |  |  |  |  |  |
| Office | -0.34 | -0.05 | 0.11 | 0.0001 |  |
| 24h | -0.31 | -0.06 | 0.08 | 0.001 |  |
| Day | -0.28 | -0.05 | 0.07 | 0.003 |  |
| Night | -0.30 | -0.05 | 0.08 | 0.001 |  |
| **Pulse pressure** |  |  |  |  |  |
| Office | -0.32 | -0.06 | 0.09 | 0.0002 |  |
| 24h | -0.31 | -0.07 | 0.08 | 0.001 | -0.07 |
| Day | -0.31 | -0.06 | 0.07 | 0.003 |  |
| Night | -0.30 | -0.06 | 0.08 | 0.002 |  |
| **Sphygmocor** |  |  |  |  |  |
| Central SBP | -0.30 | -0.04 | 0.08 | 0.006 |  |
| Central pulse pressure | -0.26 | -0.04 | 0.06 | 0.02 |  |
| Femoral pulse wave velocity | -0.29 | -0.3 | 0.07 | 0.006 |  |
| **Renal ultrasound** |  |  |  |  |  |
| Mean resistive index | -0.30 | -12.6 | 0.08 | 0.001 |  |
|  |  |  |  |  |  |
|  |  |  |  |  |  |
| **eGFR** | 0.24 | 0.03 | 0.05 | 0.005 |  |
| **Urine albumin/creatinine ratio** | -0.31 | -0.008 | 0.09 | <0.0001 | -0.02 |
| **Plasma sodium** | 0.19 | 0.2 | 0.03 | 0.047 |  |
| **Plasma potassium** | -0.22 | -1.2 | 0.04 | 0.02 |  |

**s**

**Parameters entered in the stepwise regression analysis were 24h SBP, urinary albumin/creatinine ratio, pulse wave velocity, mean renal resistive index, baseline eGFR. 24h SBP and urinary albumin/creatinine ratio were the only parameters retained. R^2^ for the stepwise regression analysis was of 0.39.**
